# Diagnostic Accuracy of Multiplex NAAT/PCR and Culture Against *Salmonella* spp.: A Comparison of Meta-Analytical Methods

**DOI:** 10.3390/pathogens15010045

**Published:** 2025-12-31

**Authors:** Xanthoula Rousou, Luis Furuya-Kanamori, Eleftherios Meletis, Olympia Lioupi, Nikolaos Solomakos, Polychronis Kostoulas, Suhail A. R. Doi

**Affiliations:** 1 Laboratory of Epidemiology, Applied Machine Learning and Biostatistics, Faculty of Public and One Health, School of Health Sciences, University of Thessaly, 43100 Karditsa, Greece; xarousou@uth.gr (X.R.); emeletis@outlook.com (E.M.); olioupi@gmail.com (O.L.); 2 UQ Centre for Clinical Research, Faculty of Medicine, The University of Queensland, Brisbane, QLD 4006, Australia; l.furuya@uq.edu.au; 3 Faculty of Veterinary Medicine, School of Health Sciences, University of Thessaly, 43100 Karditsa, Greece; nsolom@uth.gr; 4 Department of Population Medicine, College of Medicine, QU Health, Qatar University, Doha 2713, Qatar; sdoi@qu.edu.qa

**Keywords:** *Salmonella* spp., multiplex nuclear acid amplification test, accuracy, sensitivity, specificity, Bayesian latent class, bivariate, split component synthesis

## Abstract

Background: Non-typhoidal (NT) *Salmonella* spp. constitutes a major cause of foodborne illness. Culture is the gold standard, but it is time consuming, whereas multiplex nucleic acid amplification tests (NAATs)/Polymerase Chain Reaction (PCR) offer faster detection with variable reported performance. Objectives: To compare the diagnostic accuracy of multiplex NAAT/PCR and culture for *Salmonella* spp. using various statistical models with or without a gold standard assumption. Methods: A systematic search (PubMed, Web of Science, Scopus; up to April 2024) identified 44 studies (55 comparisons). Diagnostic performance was evaluated using the frequentists bivariate model (BM) and Split Component Synthesis (SCS) and the Bayesian bivariate models (BBMs) and hierarchical summary ROC (BHSROC). Results: Across models, multiplex NAAT/PCR demonstrated high specificity (>98%) but model-dependent variability in sensitivity (85.5–94.8%), consistently substantial between study heterogeneity and threshold variation. The BM and BBM yielded a higher sensitivity estimate with narrower non-overlapping confidence intervals while SCS and BHSROC models, which are more robust to threshold differences, produced more conservative estimates with wider uncertainty. In Bayesian latent class analyses, culture remained highly accurate (Se: 97.17%, 95% CrI: 70.3–99.99; Sp: 96.06%, 95% CrI: 78.9–99.99), but with wide credible intervals indicating variation between studies, perhaps due to the different protocols used. Conclusion: Model choice affects inferred diagnostic accuracy, particularly when high heterogeneity is present. Both multiplex NAAT/PCR and culture showed high accuracy; hence, a combination of the two tests could optimise rapid diagnosis and treatment. Future research should include cost effectiveness and decision analysis to update the diagnostic algorithms.

## 1. Introduction

Non-typhoidal (NT) *Salmonella* spp. is the second most common causative agent isolated from foodborne outbreaks, following *Campylobacter* spp. [1]. In 2023, there were 18 confirmed cases per 100,000 population, with a total of 1115 outbreaks [1]. The most prevalent NT Salmonella serovar in the European Union is *Salmonella enteritidis*, which accounted for approximately 71% of the reported human cases in 2023 [1,2,3]. Other significant serovars include *S. typhimurium*, monophasic *S. typhimurium* (1,4,[5],12:i:-), *S. infantis*, and *S. coeln*. Each of these serovars has unique characteristics and implications for disease management.

Currently, culture with an enrichment step remains the gold standard for diagnosing *Salmonella* spp. infections due to its high accuracy and ability to recover live isolates [4,5,6,7,8]. However, results typically take at least 48 h to obtain [4,6]. To address this delay, many laboratories now employ culture-independent diagnostic tests (CIDTs), particularly molecular methods such as multiplex nucleic acid amplification tests (NAATs)/Polymerase Chain Reaction (PCR), as rapid first-line diagnostic tools [9]. While molecular diagnostics offer faster turnaround times, they are limited in key respects, such as the inability to isolate bacteria and distinguish between active and past infections. Moreover, different NAAT/PCR platforms use various primer sets that target distinct gene areas, and not all assays are equally sensitive to all Salmonella strains. Compounding these limitations, culture performance itself can substantively vary depending on enrichment protocols and the agar type used, as these differ in inhibitory components and recovery efficiency; studies comparing agar types (often in conjunction with enrichment) have reported sensitivities ranging from 84% to 98% and specificities from 78% to 100% [4,5,6,7,8].

These limitations become particularly critical, when discrepancies arise between culture and NAAT/PCR results. In these cases, determining whether the difference is due to missed detection by culture or a false positive from NAAT/PCR and vice versa presents a significant diagnostic challenge.

To address this issue, this meta-analysis employs a range of statistical models to estimate the diagnostic accuracy of multiplex NAAT/PCR and culture. These include both frequentist models Split Component Synthesis (SCS) and bivariate (BM), as well as Bayesian approaches, such as the Bayesian bivariate model (BBM) and the Bayesian hierarchical summary receiver operating characteristic (BHSROC) model. In frequentist frameworks, the reference test is assumed to be perfect. In contrast, Bayesian latent class models acknowledge the absence of a true gold standard by treating the actual disease status as a latent (unobserved) variable. These models integrate prior information with observed data to estimate the performance of both the index test and the reference test.

Each statistical method is based on specific assumptions and has its strengths and limitations (Table 1), which depend on several factors including data structure, study sample size, and the presence of threshold differences. Therefore, applying multiple approaches enables a more comprehensive and robust evaluation of diagnostic accuracy, reducing the dependence on any single model’s assumptions. Given the anticipated heterogeneity across studies—resulting from various NAAT/PCR brands and culture conditions—methods like SCS and BHSROC are considered well suited to handle threshold effects and are expected to yield more robust summary estimates.

The overall aim of this systematic review and meta-analysis is to estimate and compare the diagnostic accuracy of multiplex NAAT/PCR for *NT Salmonella* spp., across various statistical methodologies.

## 2. Materials and Methods

### 2.1. Search Strategy and Selection Criteria

A comprehensive literature search was performed across PubMed, Web of Science, and Scopus, for peer-reviewed articles up to and including April 2024 using combinations of the following terms: “multiplex PCR”, “culture”, “Salmonella enterica”, “gastroenteritis”, “diagnostic accuracy”, “sensitivity”, and “specificity”.

After removing duplicates, two reviewers (X.R. and E.M.) independently screened articles for inclusion, first by title and abstract, and then by full article assessment. Disagreements were resolved by a third reviewer (P.K.). We included studies that (1) involved symptomatic participants providing a stool or rectal swab specimen for testing, (2) used commercially available or laboratory-developed multiplex NAAT/PCR tests, (3) both culture and multiplex NAAT/PCR were performed on the same specimen, (4) evaluated multiplex NAAT/PCR against culture for the detection of *NT Salmonella* spp., (5) provided sufficient data to construct 2 × 2 contingency tables, and (6) were published in peer-reviewed journals. Studies were excluded if they lacked a reference standard, used more than one sample per patient, were not in English, or involved non-human samples.

### 2.2. Data Extraction and Quality Assessment

Two independent reviewers (X.R. and E.M.) extracted data on study characteristics, sample size, test method characteristics, diagnostic performance metrics (true positives, false positives, false negatives, true negatives), and study design. Importantly, all meta-analyses were based on the raw cross-tabulation of multiplex NAAT/PCR against culture performed on the same specimen, as reported by each study. If studies reported the use of a third test (e.g., additional PCR, sequencing) or retesting to adjudicate discordant results, this information was extracted but adjudicated results were not used to reclassify outcomes or to modify extracted 2 × 2 tables. Discrepancies between reviewers were resolved by consensus. The methodological quality of each study was assessed using the QUADAS-2 tool [19].

### 2.3. Statistical Analysis

Various methods have been developed for the joint estimation of Se and Sp [10,12,14,17,20], and the characteristics as well as the advantages and disadvantages of the models used in this study are summarised in Table 1.

The most widely used statistical method [21] is the bivariate model that was introduced by Reitsma et al. [12] and modified by Chu et al. to account for zero cells, by using an exact binomial model instead of a continuity correction [13]. The bivariate model (BM) can also be computed via Bayesian inference (BBM) [14], thereby incorporating prior knowledge or relaxing the assumption of an imperfect standard. The BM and BBM also take into account the dependence between Se and Sp via a variance–covariance matrix [14].

The split component synthesis (SCS) method, as described in [10,11], utilises quasi-likelihood to address the issues of overdispersion commonly associated with this type of data, making it a more flexible model than the BM. In addition, high heterogeneity is frequently anticipated in these studies due to the different cut-off values that each study might use (threshold effect), for which the SCS method might be more resilient than the BM [10,22,23]. This method estimates the DOR, which is then split into the logit-transformed Se and Sp, and the summary receiver operating characteristic (SROC) curve is produced.

The threshold effect can also be dealt with by using the Bayesian hierarchical summary receiver operating characteristic (BHSROC) model, developed by Rutter and Gastonis [17] and updated by Dendukuri et al. [18]. This model addresses heterogeneity both between- and within-study levels, by incorporating in the model a threshold parameter [17,18,24].

To estimate the diagnostic accuracy of culture without assuming it as the perfect gold standard, we applied the Bayesian HSROC and BBM models under a latent class framework. In these models, neither test was assumed to be perfect, and latent disease status was inferred from the data.

Analyses were conducted in three stages. First, in the primary meta-analysis, culture was treated as the gold standard and multiplex NAAT/PCR accuracy was evaluated using BM, SCS, and the gold-standard parameterisations of BBM and BHSROC. Second, to relax the perfect-reference assumption, we fitted Bayesian latent class BBM and BHSROC models, in which true Salmonella infection status is unobserved, and the accuracy of multiplex NAAT/PCR and culture were jointly estimated. Third, we carried out a subgroup analysis using the latent class BHSROC to evaluate several factors that might influence accuracy of multiplex NAAT/PCR and culture as well as to assess heterogeneity. For the Bayesian models, sensitivity analysis was also performed to investigate the influence of priors on the accuracy estimates and also whether the two tests were conditionally dependent.

#### 2.3.1. Software

For the BM, we utilised the shiny apps Meta-Disc 2.0 [25] and MetaDTA [15,26,27]. We performed the SCS method using the SCSMeta function [10], and the statistical software R, version 4.2.3 [28]. Note that the SCS method can also be run with the diagma module in Stata [Furuya-Kanamori L & Doi SA. DIAGMA: Stata module for the split component synthesis method of diagnostic meta-analysis (RePEc:boc:bocode:s458815). <https://ideas.repec.org/c/boc/bocode/s458815.html>, accessed on 26 December 2025].

The Bayesian analyses with BBM and BHSROC were performed using the runjag package [29] of the statistical software R (model syntax code provided in the Supplementary Materials), with 100,000 iterations in the post-burn-in phase of 15,000, different initial values for each chain, and visual inspection of trace plots to assess convergence. Summary measures include median/mean estimates as well as 95% credible intervals (CrI). Model comparison was based on the Deviance Information Criterion (DIC). The BBM was also performed via the Shiny app MetaBayesDTA, version 1.5.3 [15].

#### 2.3.2. Sensitivity Analysis

##### Bayesian Methods

We used non-informative priors, weakly informative priors (restriction of the sensitivity and specificity of culture to be above 0.3 and 0.7, respectively), and informative priors for the sensitivity and specificity of culture. The informative prior normal distributions were calculated based on three studies evaluating the performance of various agar media, which reported sensitivities ranging from 84% to 98% and specificities of 78% to 100% [4,5,6]. We also investigated conditional dependence by considering the covariance between sensitivities to be fixed at 5%, 25%, 50%, or 80% of its maximum value, as illustrated previously [14].

##### Subgroup Analysis

The Bayesian HSROC latent class model was utilised for the simultaneous meta-analysis of various subgroups in investigating potential sources of heterogeneity and differences in accuracy of the multiplex NAAT/PCR. We also evaluated if there were different estimates of culture accuracy depending on the type of agar or on the transport medium used.

The factors “multiplex NAAT/PCR brand” and “manufacturer funding” were used in the subgroup analysis to explore potential sources of heterogeneity for the estimation of accuracy of multiplex NAAT/PCR. The different NAAT/PCR brands were grouped into six categories (FilmArray, Luminex, Allplex/Seeplex, BDMax, Laboratory Developed-LD- and other commercial NAAT/PCR brands). NAAT/PCR brands that could not be solely evaluated due to a small number of studies (<4 studies) were collapsed into the other commercial NAAT/PCR brands category.

The accuracy of culture was assessed regarding the agar type (HE, XLD, SS, and the use of more than one type), and the use of transport medium (no, yes, unclear). The bivariate version of I^2^ developed by [30] and also calculated by the shiny app Meta-Disc was used to calculate the overall heterogeneity since it takes into account both sensitivity and specificity as well as the correlation between them.

## 3. Results

### 3.1. Literature Search

The literature search identified, after excluding duplicates, 4720 articles for title and abstract screening, from which 186 were selected for full-text review, yielding 44 eligible studies (Supplemental Figure S1). For the primary meta-analysis, we included 45 studies with 55 comparisons between the index test and culture.

### 3.2. Study Characteristics

The characteristics of the studies published from 2009 to 2024 are presented in Table 2 and Table 3. Luminex was the most commonly evaluated index test (n = 14, 29.8%), followed by FilmArray (n = 7, 14.9%), Allplex/Seeplex (n = 6, 12.8%), and BDMax (n = 5, 11.1%). Most studies (n = 36, 80%) reported the age of the participants with the majority (n = 28, 59.57%) to include patients of all ages. Six studies (12.8%) employed only paediatric patients, and two (4.2%) only adults. Most studies were prospective, with only seven being retrospective and five using both designs. Almost all studies included patients with symptoms of gastroenteritis, and only two studies did not mention any information. Fourteen (31.11%) and twenty (44%) studies reported whether they respectively received or did not receive industry funding. Thirty-eight studies provided information on the procedure followed for culture, of which thirty-four included an enrichment step and fifteen used more than one type of agar. In total, 31% (n = 14) of the studies reported using Cary–Blair or other transport medium in all (n = 9, 20%) or in some (n = 6, 13%) of the samples. Discrepancy analysis using a third test, usually another NAAT/PCR with different primers targeting different genes and/or sequencing, was reported in 32/55 comparisons (58%); only in 12/55 (21.8%) comparisons were the results of the third test documented (Table 3).

### 3.3. Risk of Bias Analysis (QUADAS 2 Tool)

The risk of bias analysis, using the QUADAS-2 tool, is presented in Tables S3 and S4 as well as in Figure S1. All studies ranked high, indicating correct answers to most questions, and thus low risk of bias. However, several studies received funding from the manufacturer of the index test, which may introduce a serious bias. In addition, the absence of an enrichment step in four studies could pose an important risk factor influencing the overall accuracy. For several studies, the risk of bias was unclear due to limited or no information regarding the exclusion or inclusion criteria for the selection of patients for, e.g., exclusion of travellers and if prior antibiotic use, as well as culture protocol. The applicability concerns regarding the methodological framework applied for the reference standard were unclear for six studies, since no information was reported.

### 3.4. Overall Analysis

**Figure 1:**
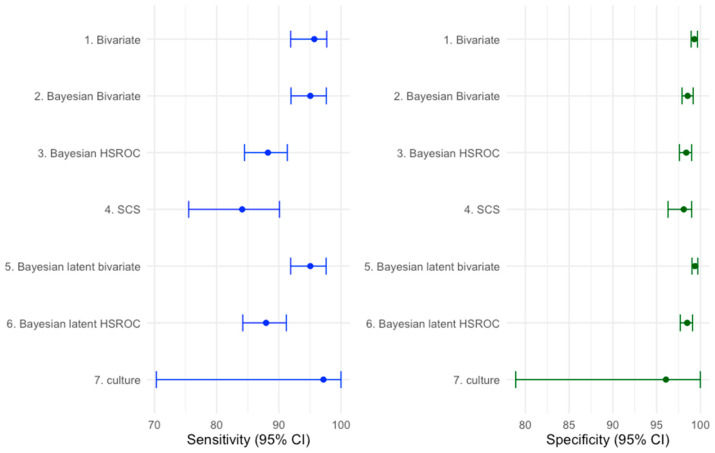
Sensitivity and specificity with 95% confidence/credible intervals (CI) (error bars) for multiplex NAAT/PCR and culture across several meta-analytic methods. Frequentist models are summarised with 95% confidence intervals (CI), whereas Bayesian models are summarised with 95% credible intervals (CrI).

The overall accuracies of multiplex NAAT/PCR and culture as estimated by the various statistical methods, are shown in Figure 1. The overall accuracies of multiplex NAAT/PCR and culture as estimated by the various statistical methods are shown in Figure 1. Two overarching findings are apparent. First, pooled Sp of multiplex NAAT/PCR was consistently high across modelling frameworks, whereas pooled Se was more model dependent. Second, the BM/BBM produced higher Se point estimates with narrower, non-overlapping intervals compared with SCS and BHSROC. Finally, Figure 1 includes both frequentist 95% confidence intervals (CI) and Bayesian 95% credible intervals (CrI).

The pooled mean estimates of the Se and the Sp of the multiplex NAATs/PCRs, with the BHSROC model, were 87.71 (95% CrI: 84.4–90.85) and 98.54 (95% CrI: 97.9–99.17), respectively. Under the Bayesian latent HSROC model, the estimated accuracy of multiplex NAAT/PCR was similar but slightly lower (Se: 87.95%, 95% CrI: 84.23–91.19%, Sp: 98.49%, 95%, CrI: 97.72–99.19), while culture showed high sensitivity and slightly lower specificity (Se: 97.17%, 95% CrI: 70.30–99.99; Sp: 96.06%, 95% CrI: 78.90–99.99). The wide credible intervals for culture, especially in sensitivity, indicate substantial uncertainty and likely protocol dependence.

### 3.5. Subgroup Analysis

Table 4 and Table 5 present latent class BHSROC subgroup estimates aimed at exploring potential sources of heterogeneity. Across multiplex NAAT/PCR platform categories, pooled Sp remained uniformly high, whereas Se varied numerically across categories. However, credible intervals were wide and overlapped substantially, indicating that the available evidence is insufficient to support definitive platform differences. Subgroup analyses by manufacturer funding status and transport medium did not show clear shifts in pooled accuracy, but these findings should be interpreted cautiously because such study-level covariates are prone to incomplete reporting.

For culture, sufficient data were available to explore agar type. The Se and Sp estimates of culture were broadly comparable across individual agar types (HE, XLD, SS), with overlapping 95% credible intervals. The “more than one agar type” subgroup showed a slightly higher Se point estimate; however, credible intervals remained wide across agar-defined subgroups, reflecting between-study variability and limited precision within subgroups.

### 3.6. Conditional Dependence Model and Informative Priors

The use of informative priors did not influence the accuracy of the index test but lowered the Se and Sp of culture (Table S4). The inclusion of a covariate to account for the conditional dependence between the sensitivities did not exceedingly alter the accuracy estimates, with a slight reduction as the percentage of dependence increased (Table S5).

## 4. Discussion

This meta-analysis comprehensively evaluates the diagnostic accuracy of multiplex NAAT/PCR versus routine culture for detecting *NT Salmonella* spp., applying a range of advanced statistical models that differ in their handling of between-study heterogeneity, and the assumption of a perfect reference standard. Across all statistical approaches, multiplex NAAT/PCR demonstrated consistently high Sp, whereas pooled Se was more model dependent and ranged from higher estimates under BM/BBM to lower, more robust estimates under SCS and BHSROC models. A previous meta-analysis [75], implementing the BM, reported similar estimates depending on the assay platform, supporting the consistency of these frequentist results under a perfect reference assumption.

The observed divergence between models could be expected because of model differences in parameterisation. BM/BBM jointly model logit Se and logit Sp without an explicit study-specific threshold parameter; when heterogeneity is high, possibly due to study variations in positivity thresholds and laboratory workflows, BM/BBM can produce overconfident pooled Se and comparatively narrower intervals, as shown in simulation studies [10,22,23]. In contrast, SCS is comparatively robust to overdispersion, because it uses a quasi-likelihood approach and BHSROC decomposes test performance into accuracy and threshold components; therefore, these approaches tend to produce more conservative Se estimates when threshold heterogeneity is high. Overall, BM/BBM estimates can be interpreted as best case accuracy under a gold standard assumption and a relatively homogeneous group. When the threshold effect is expected to be high as is often the case, then the estimates from SCS/BHSROC—particularly under a latent class framework—are more consistent with the underlying diagnostic process, and the wider intervals should be viewed as a realistic representation of uncertainty rather than a limitation of the method.

Substantial heterogeneity, in our study, likely reflects both methodological and clinical variation across studies, including differences in patient spectrum (age, disease severity, and pre-test probability), specimen handling (e.g., transport medium), and laboratory workflows (e.g., enrichment broths, agar type, number of agar types used, incubation conditions). In our dataset, at least six commercial multiplex platforms were represented, each potentially differing in gene targets, and positivity thresholds. In addition, three different agar types, with differing performance characteristics [4,5,6,7,8], could be identified. These factors alone can generate considerable heterogeneity and help explain the discrepancies between statistical methods. However, even when analyses were stratified by test brand or culture agar type, substantial heterogeneity persisted, as indicated by the wide credible intervals, suggesting that additional study-level factors—such as patient selection, disease severity, specimen transport and storage, or laboratory workflow—also influence observed accuracy. Additional simulation studies that vary in threshold effect, prevalence, sample size and degree of reference-standard imperfection, could be valuable to clarify under which conditions different meta-analytic models provide robust estimates of test performance.

The Bayesian HSROC model estimated higher Se and lower Sp of culture compared to multiplex NAAT/PCR. This pattern is supported by individual studies, particularly when culture is preceded by overnight enrichment [9,42,62]. In the study by Yoo et al. [72], three different brands of multiplex NAAT/PCR were evaluated against enriched culture; all demonstrated lower Se than culture, and one assay (Luminex) exhibited markedly poor Sp. Similarly, in a large prospective study of 6372 sequential stool samples from 5619 patients [9], the Se of multiplex NAAT/PCR was 89%, similar to what was found in our study. These patterns were broadly consistent when studies were stratified by index test brand, manufacturer funding, transport medium, and by culture agar type (HE, XLD, SS, or use of multiple agars), indicating that, within this heterogeneous evidence base, enrichment culture tends to be more sensitive but slightly less specific than multiplex NAAT/PCR.

From a clinical perspective, the high Sp and overall high Se of multiplex NAAT/PCR support its use as a rapid front-line test to accelerate diagnosis and inform early clinical management. However, NAAT/PCR as a stand-alone test with total replacement of culture has important implications for epidemiological data recording, because as already stated, NAAT/PCR does not provide an isolate for antimicrobial susceptibility testing and epidemiological surveillance. To mitigate this, laboratories may perform culture in parallel with NAAT/PCR or adopt reflex culture of NAAT/PCR-positive specimens; decision-analytic modelling (e.g., a decision tree) could be used to compare these strategies.

This meta-analysis has several strengths, including a systematic literature search of three large databases and the use of both frequentist and Bayesian methods, allowing for direct comparison of models that assume a perfect reference standard with those that do not. Nonetheless, it also has limitations. The literature search did not explicitly include grey literature or additional databases, which might have identified further studies but at the potential cost of lower methodological quality. Exploration of heterogeneity was restricted to parameters consistently reported across studies, such as index test brand and agar type; it was not feasible to examine the influence of other clinically relevant variables (e.g., age group) because of limited subgroup sizes. Subgroup analyses were constrained by small numbers of studies per category and sparse data, leading to wide credible intervals and limited ability to attribute heterogeneity to individual factors. Finally, applicability concerns about the reference standard were rated as unclear in six studies, because no detail on culture workflows (e.g., enrichment use, agar combinations) was given, and not even a report that standardised procedures were followed as done by other studies. This limits confidence in the transportability of pooled estimates to specific laboratory settings, particularly if it differs from the dominant workflows in the included studies.

Multiplex NAAT/PCR assays exhibit excellent Sp and high Se for detecting NT Salmonella spp., though inter-assay variability persists. Conventional culture remains highly accurate, particularly when enrichment is applied. Future work should prioritise standardisation and explicit reporting of culture protocols, as well as decision-analytic/cost-effectiveness analysis to optimise diagnostic testing algorithms.

## Figures and Tables

**Table 1:** Advantages, limitations, and recommended use of meta-analytical models applied for the estimation of the accuracy of multiplex NAAT/PCR and culture (when assumed as imperfect gold standard), against *NT Salmonella* spp.

Model	Strengths	Key Limitations	Best Use When
Split Component Synthesis (SCS) [10,11]	Robust to overdispersion; performs well with few studies; accommodates threshold-related heterogeneity	No meta-regression; does not model Se-Sp correlation explicitly sensitive to zero cells (requires continuity correction)	Small meta-analyses threshold effect quick synthesis when overdispersion is present normality of the data is questionable
Bivariate Model (Frequentist) [12,13]	Standard approach; allows meta-regression; models Se–Sp correlation; available shiny app; handles zero cells (exact binomial model by Chu and Cole [13])	Sensitive to zero cells if Reitsma model [12] is used; convergence issues in small datasets; assumes normally distributed logits; overconfident under high heterogeneity/threshold effect	Large dataset; no or modest threshold effect; need covariate adjustment available applications/metaDisc and MetaDTA
Bayesian Bivariate Model [14]	Handles zero cells; allows priors; supports covariates; can incorporate imperfect reference/conditional dependence; available shiny app	Computationally intensive (requires MCMC), however to a lesser extend when using the available shinny apps; usually assumes normally distributed logits	Small number of studies; use of imperfect reference standard; use of multiple reference standards; need for covariate adjustment; test for conditional dependence available applications: MetaBayesDTA [15] and bayesdta [16]
Bayesian HSROC (BHSROC) [17,18]	Explicitly models threshold heterogeneity (threshold parameter); sROC curve; allows priors; supports covariates; can incorporate imperfect reference/conditional dependence; more robust to non-normality	More complex model and parameterisation; computationally intensive (requires MCMC); requires careful prior/model specification; no available applications; usually assumes normally distributed logits	Substantial heterogeneity (threshold effect); use of imperfect reference standard; use of multiple reference standards; can include covariates; test conditional dependence

**Table 2:** Characteristics of the included studies *.

Study	Sampling Period (MM/YY)	Study Design	Number of Patients	Participants	Age Category
Ahmed A.O. et al. 2024 [31]	02/2020–10/2020	Prospective	50	Secretory diarrhoea (acute and persistent)	Paediatric and adult
Bessede E. et al. 2011 [32]	15/06/2009–30/10/2009	Prospective	242	Gastrointestinal illness, hospitalised for less than 48 h	Paediatric and adult
Buchan B.W. et al. 2013 [33]	2007–2011	Retrospective	105	Routine testing 4 US laboratories	Paediatric and adult
Buchan B.W. et al. 2013 [33]	07/2011–11/2011	Prospective	1139	Routine testing 4 US laboratories	Paediatric and adult
Buss et al. 2015 [34]	05/2013–09/2013	Prospective	1556	Routine testing; 4 geographically distinct sites	Paediatric and adult
Claas et al. 2013 [35]	02/2010–10/2010	Prospective	489	Routine testing; 4 countries	Paediatric and adult
Coupland L.J. et al. 2013 [36]	NR	Retrospective	201	Semi-formed or liquid faecal samples	Paediatric and adult
Cybulski R.J. et al. 2018 [37]	01/2017–09/2017	Prospective	1887	Symptoms of gastroenteritis, newly-admitted (<3 days) inpatients and outpatients; 17 outpatient clinics	Paediatric and adult
Deng J. et al. 2015 [38]	11/2012–05/2013	Prospective	290	Diarrhoeal faecal samples submitted to Zhujiang Hospital	Paediatric and adult
Dror S.K. et al. 2016 [39]	11/2013–04/2014	Retrospective two regional laboratories	161	Frozen stool bank; two regional laboratories	NR
Dror S.K. et al. 2016 [39]	11/2013–04/2014	Prospective	94	NR	NR
Duong V.T. et al. 2016 [40]	2009–2014	NR	479	Diarrhoeal disease; 3 hospitals; 3 watery or loose stools within 24 h or one episode of bloody and/or mucoid diarrhoea	Paediatric and adult
Halligan E. et al. 2014 [41]	11/2011–07/2012	Prospective	1396	Diarrhoeal faecal samples (3 or more liquid stools in 24 h); some patients had hospital associated diarrhea	Paediatric and adult
Harrington et al. 2015 [42]	12/2012–09/2013	Prospective	2922	Soft or diarrhoeal faecal samples; 6 clinical centers in the United States, 1 in Canada, and 1 in Mexico	Paediatric and adult
Harrington et al. 2015 [42]	2007, 2013, 03/2012, 08/2013	Retrospective	618	Soft or diarrhoeal faecal samples; 6 clinical centers in the United States 1 in Canada and 1 in Mexico	Paediatric and adult
Hu Q. et al. 2014 [43]	NR	Prospective	9439	Stool specimens from diarrhoeal outpatients were collected in 11 hospitals in Shenzhen.	NR
Huang R.S.P. et al. 2016 [44]	05/2013–01/2014 and m-PCR testing in 2015	Prospective retrospective	152	Acute gastroenteritis	Paediatric
Huang Shu-Huan et al. 2018 [45]	07/2015–04/2016	Prospective	217	Symptoms of gastroenteritis	Paediatric and adult
Jo S.J. et al. 2022 [46]	10/2019–08/2020	Prospective	184	Paediatric patients with diarrhoea who visited Seoul St. Mary’s hospital. Routine laboratory tests were ordered for patients with diarrhoea that started within 72 h of presentation	Paediatric
Kellner T. et al. 2019 [47]	12/2014–03/2018	Prospective	3089	≥3 episodes of vomiting and/or diarrhoea in the preceding 24 h and <7 days of symptoms two large hospitals, emergency departments (EDs) and a provincial nursing triage telephone advice line	Paediatric
Khare R. et al. 2014 [48]	NR	Retrospective	270	Routine GI testing	NR
Khare R. et al. 2014 [48]	NR	Prospective	230	Routine GI testing	NR
Knabl L. et al. 2016 [49]	02/2015–03/2015	Prospective	893	Stool specimens of patients with diarrhoea; practitioners and hospitals (1 tertiary and 9 district hospitals)	Paediatric and adult
Knoth C. et al. 2024 [50]	01/2015–08/2017	Prospective/ retrospective	1554	Routine testing; 4 geographically different US sites; previously characterised: randomly chosen 260 positive specimens and 152 negative specimens	Paediatric and adult
Koeffer J. et al. 2024 [51]	01/2023–03/2023	Prospective	500	Acute gastroenteritis	Paediatric and adult
Koffer J. and Frontzek A. 2023 [52]	NR	Retrospective	745	Leftover samples from daily routine; 2 laboratories	
Koo S.H. et al. 2022 [53]	02/2016–10/2016	Prospective	299	Acute gastroenteritis; specimens within 72 h of patient admission	Adults
Kosai K. et al. 2021 [54]	10/2016–03/2018	Prospective	268	Clinical stool samples submitted to laboratories	
Liu J. et al. 2012 [55]	NR		205	Patients from Tanzania, Bangladesh, Pakistan; 181 were previously tested as positive	Paediatric and adult
Liu J. et al. 2013 [56]	2010–2011, Tanzania 2008–2009, Bangladesh	Retrospective	109	Clinical samples from Tanzania and Bangladesh	Paediatric and adult
Martin A. et al. 2018 [57]	01/2016–09/2016	Prospective and retrospective	394	Diarrhoeal faecal samples (consecutive). University hospital	Paediatric and adult
McAuliffe G.N. et al. 2017 [58]	06/2015–10/2015	Prospective	237	Routine testing; 1 large laboratory	Paediatric and adult
Navidad J.F. et al. 2013 [59]	06/2011–06/2012	Prospective	254	Gastroenteritis; hospitals, long-term-care facilities, child care facilities, area restaurants, and the Milwaukee refugee screening facility	Paediatric and adult
O’Leary J. et al. 2009 [60]	04/2008–06/2008	Prospective	773	Gastroenteritis (did not exclude travellers)	
Onori M. et al. 2014 [61]	04/2010–08/2011	Prospective	245	Paediatric patients, admitted for presumptive infectious diarrhoea to the Paediatric and Infectious Disease Unit of Bambino Gesù Children’s Hospital in Rome, Italy	Paediatric
Pankhurst L. et al. 2014 [62]	06/2009–09/2012		839	NR	
Pankhurst L. et al. 2014 [62]	06/2009–09/2012		948	NR	
Park K. and Shin B.-M. 2024 [63]	01/2021–07/2022	Prospective	366	Acute gastroenteritis	Adults
Patel A. et al. 2014 [64]	07/2013–12/2013	Prospective	211	Gastroenteritis	Paediatric and adult
Perry M.D. et al. 2014 [65]	one summer month 2012	Prospective	991	Diarrhoeal faecal samples	Paediatric and adult
Rintala A. et al. 2016 [66]	04/2014–06/2014	Prospective	1168	Routine testing; 2 large laboratories; 2 countries	Paediatric and adult
Roy C. et al. 2020 [67]	04/2011; 01/2019;11/2018; 03/2019	Retrospective/prospective	251	Samples collected from the Pediatric and Adult Emergency Department of the university hospital	Paediatric and adult
Tilmanne A. et al. 2019 [68]	05/2015–10/2016	Prospective, case control study	178	Acute gastroenteritis; 2 large hospitals	Paediatric
Van Lint P. et al. 2015 [69]	NR	Prospective	1687	Routine testing	
Wiemer D. et al. 2011 [70]	2007–12/2008	Prospective	393	Routine testing	Paediatric and adult
Wohlwend N. et al. 2016 [71]	07/2013–08/2013 (262) 11/2014–03/2014 (794)	Prospective	1056	NR	
Yoo J. et al. 2019 [72]	01/2016–10/2016		182	Stool samples submitted to the department of laboratory medicine in the Seoul St Mary’s Hospital, The Catholic University of Korea, Seoul, Korea	Paediatric and adult
Zhang C. et al. 2015 [73]	01/2012–12/2012	Cross sectional	122	Hospitalised children with acute diarrhoea, during routine surveillance Diarrhea Department, National Institute for Infectious Disease Control and Prevention (DD-IVDC)	Paediatric
Zhang J. et al. 2019 [74]	01/2016–09/2016 retrospective before December 2015	Prospective and retrospective	462	faecal specimens; 3 locations	Paediatric and adult

* NR: not reported.

**Table 3:** Results and characteristics regarding index test and culture methods.

Study	Discrepancy Analysis	Test *	Conflict of Interest	Transport Medium	Culture *	TP	FP	TN	FN
Ahmed A.O. et al. 2023 [31]	NR	FilmArray	No	No	XLD, SS enrichment	2	0	48	0
Bessede E. et al. 2011 [32]	NR	Allplex/ Seeplex	NR	No	NR	14	8	215	5
Buchan B.W. et al. 2013 [33]	NR	ProGastro	Yes	Yes	XLD, HE; incubated at 35 °C; 48 h	3	0	102	0
Buchan B.W. et al. 2013 [33]	All FP -> TP bidirectional sequencing	ProGastro	Yes	Yes	XLD, HE; incubated at 35 °C; 48 h	20	10	1108	1
Buss et al. 2015 [34]	rt-PCR; sequencing	FilmArray	Yes	Yes	HE or EMB GN enrichment	31	6	1519	0
Claas et al. 2013 [35]	PCR, bidirectional sequencing; primers targeting different genomic regions	Luminex	Yes	No	Standardised procedures	62	13	408	6
Coupland L.J. et al. 2013 [36]	NR	Allplex/Seeplex	No	No	XLD enrichment	16	0	182	3
Cybulski R.J. et al. 2018 [37]	NR	FilmArray	Yes	Yes	SS	13	6	1878	1
Deng J. et al. 2015 [38]	PCR; sequencing 3 FP -> TP	Luminex	No	No	SS, HE; enrichment	25	6	254	5
Dror S.K. et al. 2016 [39]	qPCR, G-DiaBact, 1 FP -> TP	NanoCHIP	No	Yes	CHROM; enrichment in MKT	13	8	140	0
Dror S.K. et al. 2016 [39]	qPCR; 1 FP -> TP	NanoCHIP	No	Yes	CHROM; enrichment in MKT	2	2	90	0
Duong V.T. et al. 2016 [40]	rtPCR; 44 FP -> TP	Luminex	No	No	XLD; SB enrichment; 37 °C overnight	38	172	267	2
Halligan E. et al. 2014 [41]	NR	Luminex	Yes	No	XLD SB enrichment before ABC Harlequin chromogenic agar	11	36	1349	0
Harrington et al. 2015 [42]	bidirectional sequencing	BD Max	Yes	Both	XLD, HE, SS, CHROM; SB or GN enrichment; 48 h at 35 °C	34	26	2857	5
Harrington et al. 2015 [42]	bidirectional sequencing; PCR	BD Max	Yes	Both	XLD, HE, SS, CHROM; SB or GN enrichment; 48 h at 35 °C	166	1	450	1
Hu Q. et al. 2014 [43]	NR	LD	No	NR	standardised procedures; enrichment	278	3	9158	0
Huang R.S.P. et al. 2016 [44]	NR	FilmArray	No	Yes	XLD, HE; incubated at 35 °C; 48 h	23	1	127	1
Huang R.S.P. et al. 2016 [44]	NR	Luminex	No	Yes	XLD, HE; incubated at 35 °C; 48 h	19	1	127	5
Huang Shu-Huan et al. 2018 [45]	NR	Luminex	No	NR	Blood agar/EMB, XLD with GN enrichment	36	8	173	0
Jo S.J. et al. 2022 [46]	BD MAX	FilmArray	NR	Yes	MacConkey; incubated in ambient air at 35 °C No enrichment	6	2	176	0
Kellner T. et al. 2019 [47]	RT-qPCR targeting a conserved region of the Salmonella invA gene	Luminex	Yes	Yes	SS, WB; SB enrichment atmospheric oxygen (35 °C, 24 h)	43	9	3025	12
Khare R. et al. 2014 [48]	rt-PCR	Luminex	NR	Yes	Standard methods	20	0	246	4
Khare R. et al. 2014 [48]	rt-PCR	FilmArray	NR	Yes	Standard methods	24	0	246	0
Khare R. et al. 2014 [48]	rt-PCR	Luminex	NR	Yes	Standard methods	1	0	229	0
Khare R. et al. 2014 [48]	rt-PCR	FilmArray	NR	Yes	Standard methods	1	1	228	0
Knabl L. et al. 2016 [49]	NR	BD Max	NR		SB enrichment-24 h; HE: 37 °C, 24 h	6	8	877	2
Knoth C. et al. 2024 [50]	Additional PCR/sequencing assays or retesting with a separate aliquot of stool	BioCode	NR	both	GN enrichment; standardised procedures	25	12	1512	5
Koeffer J. et al. 2024 [51]	NR	LD	Yes, inclusion in the acknowledgements	NR	SB enrichment: 35 °C ± 2 °C, 24 h, then CSE and XLD; 24 h, 35 °C ± 2 °C	2	0	498	0
Koffer J. and Frontzek A. 2023 [52]	Allplex PCR 3 of 3 FP, then TP	LD	NR	NR	NR	216	3	323	0
Koo S.H. et al. 2022 [53]	LD PCR assays 16 FP -> TP	BDMax	No	No	SS; SB enrichment	42	17	238	2
Kosai K. et al. 2021 [54]	Luminex for the FP results and re-examination with the Verigene for the FN results. All FN -> TP	Verigene	Yes	No	CHROM, SS No enrichment	53	1	211	3
Liu J. et al. 2012 [55]	q-PCR; all FP -> TP FN -> TN	LD	No		XLD	14	9	181	1
Liu J. et al. 2013 [56]	NR	LD	NR	No	XLD	6	0	72	0
Martin A. et al. 2018 [57]	NR	Allplex	Yes	No	SB enrichment: 35 °C ± 2 °C, 24 h SS	40	11	342	1
McAuliffe G.N. et al. 2017 [58]	BD MAX	EntericBio	No	No	XLD; 36 °C in ambient air; SB enrichment; 36 °C for 18–24 h prior to inoculation onto XLD plates	1	0	236	0
Navidad J.F. et al. 2013 [59]	m-PCR was repeated or16S DNA sequencing	Luminex	No	Both	XLD enrichment	26	0	226	2
O’Leary J. et al. 2009 [60]	NR	EntericBio	No	NR	XLD, DC, Hal1 incubation overnight at 37 °C; enrichment overnight in SB; subculture to Hal1	4	0	769	0
Onori M. et al. 2014 [61]	PCR; 6 FN -> TP	Seeplex	No	No	HE, CHROM; 35–37 °C aerobic; SB enrichment	9	0	229	7
Pankhurst L. et al. 2014 [62]	PCR; FP not enough sample to test if TP by PCR; 10 FN -> TP	Luminex	NR	NR	XLD, SB, incubated at 37 °C; 24 h; after 24 h, SB inoculated onto SALM and incubated for a further 24 h at 37 °C	15	9	797	18
Pankhurst L. et al. 2014 [62]	PCR, 2 FP -> TP 19 FN -> TP	MassCode	NR	NR	XLD, SB, incubated at 37 °C; 24 h; after 24 h, SB inoculated onto SALM and incubated for a further 24 h at 37 °C	26	16	872	34
Park K. and Shin B.-M. 2024 [63]	NR	Seeplex	NR	NR	HE, SS; No enrichment	8	19	326	0
Patel A. et al. 2014 [64]	NR	Luminex	No	NR	SS, XLD SB; GN enrichment	19	3	289	3
Perry M.D. et al. 2014 [65]	NR	Luminex	Yes	No	NR	4	3	984	0
Perry M.D. et al. 2014 [65]	NR	Savyon GIP	Yes	No	NR	3	0	984	1
Rintala A. et al. 2016 [66]	RIDA^®^GENE Bacterial Stool Panel (R-Biopharm AG, Germany)	Amplidiag	Yes	No	NR	16	4	1148	0
Roy C. et al. 2020 [67]	RIDA^®^GENE Bacterial Stool Panel (R-Biopharm AG, Germany)	Novodiag	Yes, kits provided by manufacturer	Yes, only the retrospective samples	HE; selenite–lactose broth enrichment overnight at 35 °C ambient air; second HE plate after enrichment	46	0	195	0
Tilmanne A. et al. 2019 [68]		Luminex	No	No	SS No enrichment	7	22	149	0
Van Lint P. et al. 2015 [69]	rtPCR	LD	No		SS enrichment	96	33	1556	0
Wiemer D. et al. 2011 [70]	rtPCR	LD	No	No	SS enrichment	71	1	318	3
Wohlwend N. et al. 2016 [71]	NR	BD Max	No	Both	HE, XLD; SB was subcultured after 15 h of aerobic incubation onto HE and XLD agar	14	3	1039	0
Yoo J. et al. 2019 [72]	Other m-PCRs	Luminex	Yes	No	HE incubated at 37 °C overnight; GN enrichment: GN incubated for 6 h and subcultured on HE agar	14	54	110	4
Yoo J. et al. 2019 [72]	Other m-PCRs	BDMax	Yes	No	HE incubated at 37 °C overnight; GN enrichment: GN incubated for 6 h and subcultured on HE agar	13	1	163	5
Yoo J. et al. 2019 [72]	Other m-PCRs	Seegene/Allplex	Yes	No	HE incubated at 37 °C overnight; GN enrichment: GN incubated for 6 h and subcultured on HE agar	12	4	160	6
Zhang C. et al. 2015 [73]	NR	LD	No	No	NR	3	0	119	0
Zhang J. et al. 2019 [74]	NR	FilmArray		Both	CHROM; SBGB enrichment	15	21	426	0

* Abbreviations: LD: laboratory developed multiplex NAAT/PCR, NR: not reported, TP: true positive, FP: false positive, FN: false negative, TN: true negative, SB: selenite broth, SBGB: selenite brilliant green broth, CSE: Chromid Salmonella Elite, XLD: Xylose Lysine Deoxycholate agar, SS: Salmonella Shigella agar, SALM or CHROM: Salmonella Chromogenic medium, MAC: MaConkey agar, HE: Hektoen Enteric agar, GN: Gram-Negative broth, EMB: Eosin Methylene Blue plate, MKT: Muller–Kauffmann Tetrathionate broth, DC: Desoxycholate Citrate agar, Hal1: Harlequin medium.

**Table 4:** Sensitivity and specificity estimate with 95% credible intervals (CrI) calculated by the latent BHSROC model, for the different categories of multiplex NAAT/PCR (brands FilmArray, Luminex, BDMax, Allplex/Seeplex, other brands, laboratory developed-LD-multiplex NAAT/PCR), and for funding or not of all or part of the research by the manufacturer.

Subgroup	Sensitivity (95% CrI)	Specificity (95% CrI)	Studies (n)
Laboratory developed			
Yes	88.51 (63.84, 98.39)	96.96 (83.25, 99.99)	7
No	87.33 (83.3, 90.56)	98.65 (97.9, 99.28)	38
culture	96.86 (70.56, 99.98)	94.5 (66.27, 99.99)	
Index test brand			
FilmArray	83.19 (60.07, 95.71)	98.08 (94.77, 99.79)	7
Luminex	87.71 (79.87, 94.07)	97.99 (94.77, 99.73)	14
BDMax	85.5 (80.4, 96.68)	97.85 (91.11, 99.99)	5
Allplex/Seeplex	81.01 (58.22, 95.67)	98.48 (92.74, 99.999)	6
Laboratory developed	88.45 (63.8, 98.12)	96.98 (82.89, 99.99)	8
Other commercial NAAT/PCRs	83.62 (69.51, 93.09)	98.87 (96.52, 99.92)	7
culture	96.82 (69.33, 99.99)	97.26 (78.39, 99.998)	
Manufacturer funded			
Yes	87.77 (81.3, 92.45)	98.3 (96.62, 99.39)	13
No	89.69 (83.33, 94.51)	97.88 (94.67, 99.48)	20
No information	79.15 (63.19, 89.45)	98.8 (97.5, 99.72)	11
culture	97.04 (70.83, 99.98)	94.51 (66.38, 99.998)	
Transport medium			
Yes	85.79 (77.79, 91.72)	98.57 (97.37, 99.43)	11
No	85.796 (80.17, 90.56)	98.8 (97.52, 99.8)	19
No information	88.02 (71.29, 97.02)	97.1 (93.15, 99.33)	14
culture	97.14 (70.88, 99.98)	94.37(66.33, 99.998)	

**Table 5:** Sensitivity and specificity estimate with 95% credible intervals (CrI) calculated by latent BHSROC model, for the different agar types used for culture and for the use or not of a transport medium.

Culture Agar Type *	Sensitivity (95% CrI) *	Specificity (95% CrI) *	Studies (n)
HE	95.23 (64.94, 99,99)	98.33 (80.39, 99.99)	3
XLD	95.81 (66.07, 99,98)	98.14 (79.87, 99.99)	6
SS	94.35 (63.58, 99.98)	97.88 (76.61, 99.998)	5
more than one type	96.46 (68.29, 99.97)	97.07 (75.23, 99.99)	13
Overall index test accuracy	87.68 (81.8, 92.42)	98.45 (97.12, 99.42)	

* Abbreviations: CrI: credible interval, XLD: Xylose Lysine Deoxycholate agar, SS: Salmonella Shigella agar, HE: Hektoen Enteric agar.

## Data Availability

All data generated or analysed during this study are included in this published article (and its Supplementary Information Files).

## Supplementary Materials

The following supporting information can be downloaded at https://www.mdpi.com/article/10.3390/pathogens15010045/s1, Figure S1: PRISMA flow diagram of the systematic process for the selection of the relevant studies [76]; Figure S2: The percentage of studies with low high or unclear (a) risk of bias and (b) concerns of applicability; Figure S3: Hierarchical summary ROC curve; Table S1. Signaling questions used in the risk of bias analysis (QUADAS 2 tool); Table S2. Rank of studies based on the QUADAS tool analysis; Tables S3. Prior distributions for the Bayesian models with or without gold standard; Table S4. Results from the Bayesian hierarchical latent class (LC-BHSROC) model; Table S5. Results from the conditional dependence Bayesian hierarchical latent (LC-BHSROC) model with weakly informative priors for the reference test.
