# Supplementary material for: Diagnostic Accuracy of Multiplex NAAT/PCR and Culture Against Salmonella spp.: A Comparison of Meta-Analytical Methods
Source: Pathogens. 2025 Dec 31;15(1):45. doi: 10.3390/pathogens15010045 (PMC12845073; doi:10.3390/pathogens15010045)
Supplement: Supplementary file 1 [file pathogens-15-00045-s001.zip › pathogens-4050194-SI.pdf]

Supplementary material

Figure S1: PRISMA flow diagram of the systematic process for the selection of the relevant studies(1)

Figure S2: The percentage of studies with low high or unclear a) risk of bias and b) concerns of applicability

Figure S3. Hierarchical Summary ROC curve

Table S1. Signaling questions used in the risk of bias analysis (QUADAS 2 tool)

Table S2. Rank of studies based on the QUADAS tool analysis

Table S3. Prior distributions for the Bayesian models with or without gold standard

Table S4. Results from the Bayesian hierarchical latent class (LC-BHSROC) model

Table S5. Results from the conditional dependence Bayesian hierarchical latent (LC-BHSROC) model with weakly informative priors for the reference test.

***1* Model code for rjags-** Bayesian latent hierarchical (BHSROC) model

2. Model code for rjags-Bayesian latent bivariate

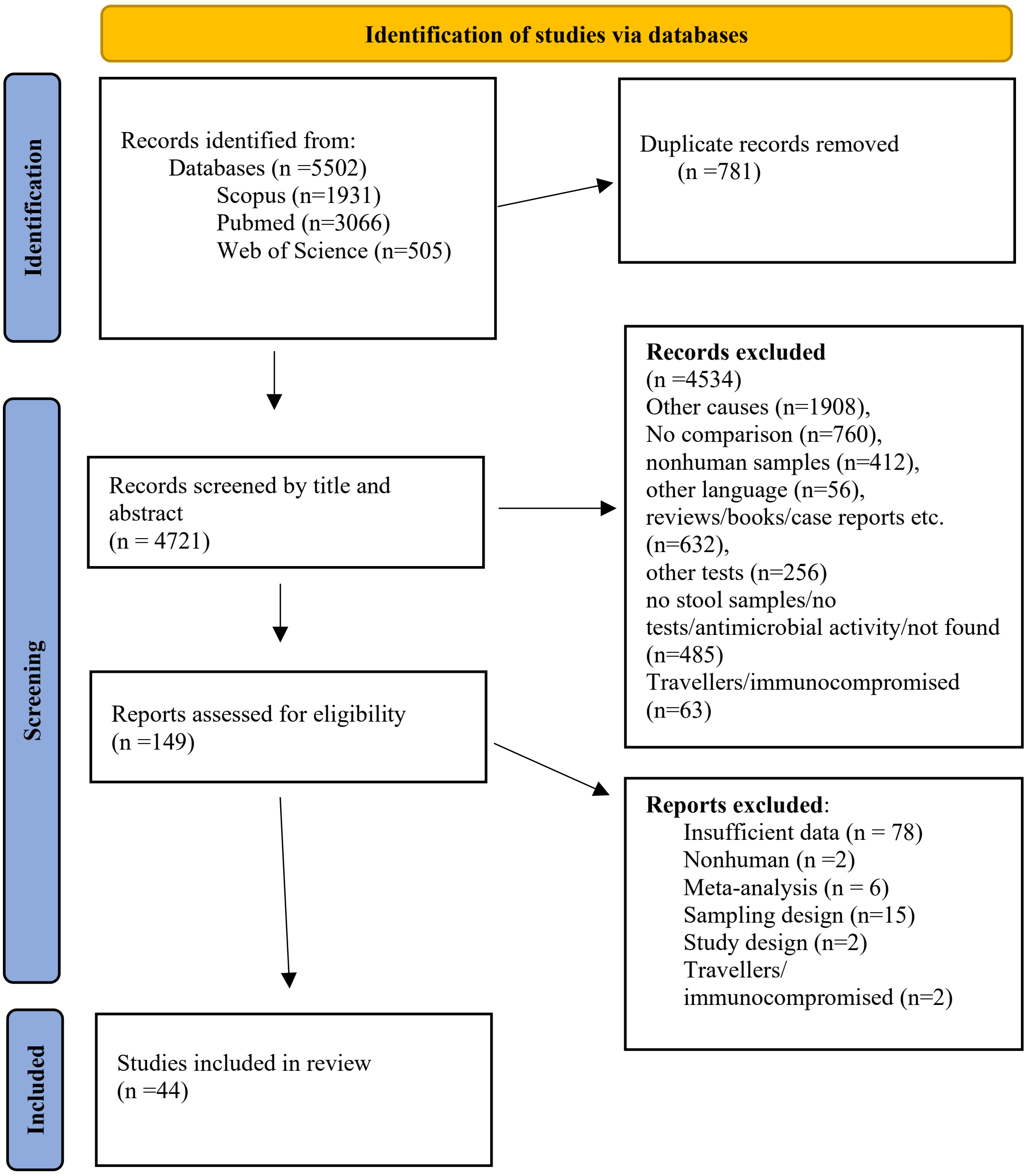

Figure S2: PRISMA flow diagram of the systematic process for the selection of the relevant studies

Table S1. Signaling questions used in the risk of bias analysis (QUADAS 2 tool)

Domain	Patient Selection	Index Test	Reference Standard	Flow and Timing
**Signalling questions (risk of bias) (yes/no/unclear)**	Was a consecutive or random sample of patients enrolled?	Were the index test results interpreted without knowledge of the results of the reference standard?	Is the reference standard likely to correctly classify the target condition?	Was there an appropriate interval between index test(s) and reference standard?
	Was a case-control design avoided?	If a threshold was used, was it pre-specified?	Were the reference standard results interpreted without knowledge of the results of the index test?	Did all patients receive a reference standard?
	Did the study avoid inappropriate exclusions?	Did all patients receive the index test?	Was the procedure followed based on specific guidelines?	Did all patients receive the same reference standard?
	Was the selection of samples **not** based on positivity by culture methods? (frozen stool bank) (retrospective design)	Was the procedure followed, strictly based on manufacturer's instructions?	Were transport media used?	Were all patients included in the analysis?
		Was the study funded by the manufacturer?	Was an enrichment step used?	
**Concerns regarding applicability: High/low/unclear**	If the samples are only from travelers, then the concerns are high	Was the index test used a laboratory developed test with information on the reagents and sources? If yes the concern is low	If the procedure followed was based on specific guidelines, then the concern is low	

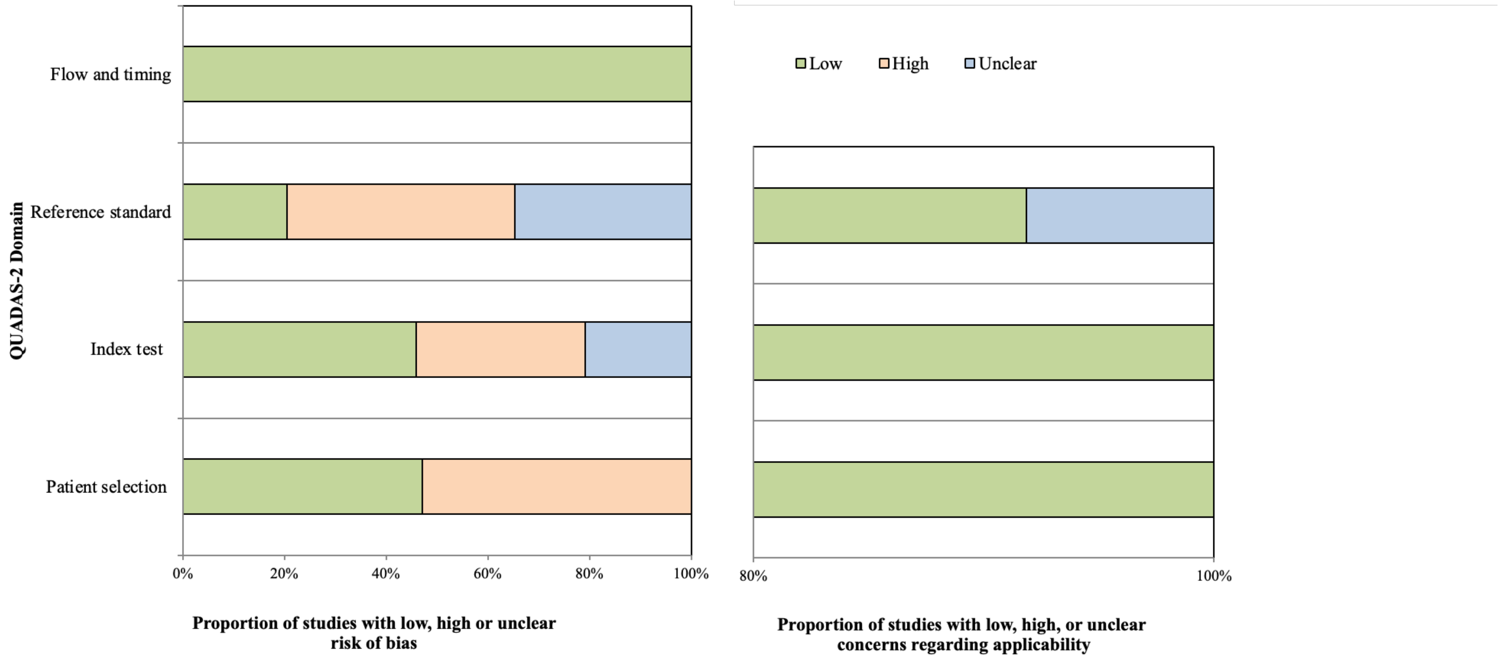

Figure S2: The percentage of studies with low high or unclear a) risk of bias and b) concerns of applicability

Table S2. Rank of studies based on the QUADAS tool analysis

Studies	Patient selection	Index test	Reference standard	Flow and Timing	Number of questions with a “yes” answer	Rank
Ahmed A.O. et al. 2023(2)	4	5	4	4	17	0.94
Bessede E. et al 2011(3)	4	4	3	4	15	0.83
Buchan B.W. et al 2013 prospective(4)	4	4	5	4	17	0.94
Buchan B.W. et al 2013 retrospective(4)	3	4	4	4	15	0.83
Buss S.N. et al 2015(5)	4	4	5	4	17	0.94
Claas et al 2013(6)	4	4	3	4	15	0.83
Coupland L.J. et al 2013(7)	4	5	4	4	17	0.94
Cybulski R.J. et al 2018(8)	4	4	5	4	17	0.94
Deng J. et al 2015 (9)	4	5	4	4	17	0.94
Dror S.K. et al 2016 prospective(10)	4	5	4	4	17	0.94
Dror S.K. et al 2016 retrospective(10)	3	5	5	4	17	0.94
Duong V.T. et al 2016(11)	3	5	4	4	16	0.89
Halligan E. et al 2014(12)	4	4	4	4	16	0.89
Harrington et al 2015 prospective(13)	4	4	5	4	17	0.94
Harrington et al 2015 retrospective(13)	3	4	5	4	16	0.89
Hu Q. et al 2014 (14)	4	5	4	4	17	0.94
Huang R.S. et al 2016 (15)	3	5	4	4	16	0.89
Huang Shu-Huan et al 2018(16)	4	5	4	4	17	0.94
Jo S.J. et al. 2022(17)	4	4	4	4	16	0.89
Kellner T. et al 2019(18)	4	4	5	4	17	0.94
Khare R. et al. 2014(19)	4	4	4	4	16	0.89

Knabl L. et al 2016(20)	4	4	4	4	16	0.89
Knoth C. et al. 2024(21)	4	4	3	4	15	0.83
Koeffer J. et al. 2024(22)	4	4	4	4	16	0.89
Koffer J. and Frontzek A. 2023(23)	4	4	3	4	15	0.83
Koo S.H. et al. 2022(24)	4	5	4	4	17	0.94
Kosai K. et al 2021(25)	4	4	3	4	15	0.83
Liu J. et al. 2011(26)	3	5	3	4	15	0.83
Liu J. et al. 2012(27)	4	5	3	4	16	0.89
Martin A. et al 2018(28)	3	4	4	4	15	0.83
McAuliffe G.N. et al 2017(29)	4	5	4	4	17	0.94
Navidad J.F. et al 2013(30)	4	5	3	4	16	0.89
O'Leary J. et al 2009(31)	4	5	4	4	17	0.94
Onori M. et al 2014(32)	4	5	4	4	17	0.94
Pankhurst L. et al 2014 retrospective(33)	4	4	4	4	16	0.89
Pankhurst L. et al 2014 retrospective(33)	4	4	4	4	16	0.89
Park K. and Shin B.-M. 2024(34)	4	4	3	4	15	0.83
Patel A. et al 2014(35)	4	5	4	4	17	0.94
Perry M.D. et al 2014(36)	4	4	3	4	15	0.83
Rintala A. et al 2016(37)	4	4	3	4	15	0.83
Roy C. et al 2020(38)	3	4	5	4	16	0.89
Tilmanne A. et al 2019(39)	3	5	3	4	15	0.83
Van Lint P. et al. 2015(40)	4	5	4	4	17	0.94
Wiemer D. et al 2011(41)	4	5	4	4	17	0.94
Wohlwend N. et al 2016(42)	4	5	4	4	17	0.94
Yoo J. et al.. 2019(43)	4	4	4	4	16	0.89

Zhang J. et al. 2015(44)	4	5	3	4	16	0.89
Zhang J. et al. 2019(45)	4	4	3	4	15	0.83

Table S3. Prior distributions for the Bayesian models with or without gold standard

Parameter	Hierarchical distribution	Prior distribution
p_i		*beta (1,1)*
BHSROC		
α_i	$\sim N(\Lambda, \sigma_1)$	$\Lambda \sim U(-6,6)$<br>$s_1 \sim dgamma(4,2)$
θ_i	$\sim N(\Theta, \sigma_2)$	$\Theta \sim U(-10,10)$<br>$s_2 \sim dgamma(4,2)$
β		$U(-2,2)$
$\mu_{seculture}$	$\sim N(ms_2, \sigma_{se})$	$ms_2 \sim dnorm(0,0.25)$
$\mu_{spculture}$	$\sim N(mc_2, \sigma_{sp})$	$mc_2 \sim dnorm(0,0.25)$
Bivariate		
μ_{se}	$\sim N(m_1, \sigma_{se})$	$m_1 \sim dnorm(0,0.25)$
μ_{sp}	$\sim N(m_2, \sigma_{sp})$	$m_2 \sim dnorm(0,0.25)$
σ_{se},, σ_{sp}		$s_{se}, s_{sp} \sim dgamma\ (2,0.5)$
r		$r \sim U(-1,1)$

Table S4. Results from the Bayesian hierarchical latent class (LC-BHSROC) model

**Parameter Estimates**	**Model- Conditional independence**
	**mean % (95%CI)**
Non-informative priors	
Sensitivity (*Multiplex PCR*)	88.03 (84.42,90.898)
Specificity (*Multiplex PCR)*	98.4993 (97.76,99.12)
Sensitivity *(culture)*	97,05 (61.95, 99.99)
Specificity *(culture)*	89.77 (99.98,1)
Restricted priors for reference test	
Sensitivity (*Multiplex PCR*)	88.07 (84.35,90.96)
Specificity (*Multiplex PCR*)	98,49 (97.77,99.14)
Sensitivity *(culture)*	97.21 (70.8,99.98)
Specificity *(culture)*	95.52 (74.15,99.998)
Informative priors for:<br>***sensitivity of culture (mean=93.3%, 95%CI: 83.9%,98%)***<br>***specificity of culture (mean=94.4%, 95%CI: 78%,99.5%)***	
Sensitivity (*Multiplex PCR*)	88.05 (84.49,91.15)
Specificity (*Multiplex PCR*)	98.49 (97.72,99.11)
Sensitivity *(culture)*	95.92 (90.05,99.06)
Specificity *(culture)*	98.19 (90.5,99.98)

Table S5. Results from the conditional dependence Bayesian hierarchical latent (LC-BHSROC) model with weakly informative priors for the reference test.

**Model-Conditional dependence**	**mean % (95%CI)**
5% of the maximum possible covariance	
Sensitivity (Multiplex PCR)	88.04 (84.47,91.02)
Specificity (Multiplex PCR)	98.5 (97.76,99.11)
Sensitivity (culture)	97.14 (69.63, 99.98)
Specificity (culture)	95.27 (73.96,99.998)
25% of the maximum possible covariance	
Sensitivity (Multiplex PCR)	87,87 (84.27, 90.898)
Specificity (Multiplex PCR)	98,55 (97.82, 99.15)
Sensitivity (culture)	96.78 (70.49,99.98)
Specificity (culture)	95.48 (73.96,99.998)
50% of the maximum possible covariance	
Sensitivity (Multiplex PCR)	87,6 (84.297, 90.64)
Specificity (Multiplex PCR)	98.66 (97.94, 99.22)
Sensitivity (culture)	95.91 (69.24, 99.92)
Specificity (culture)	95.48 (74.08, 99.998)
80% of the maximum possible covariance	
Sensitivity (Multiplex PCR)	86.398 (83.05, 89.3)
Specificity (Multiplex PCR)	98.97 (98.38, 99.48)
Sensitivity (culture)	92.77 (63.48, 99.84)
Specificity (culture)	95.33 (74.06,99.997)

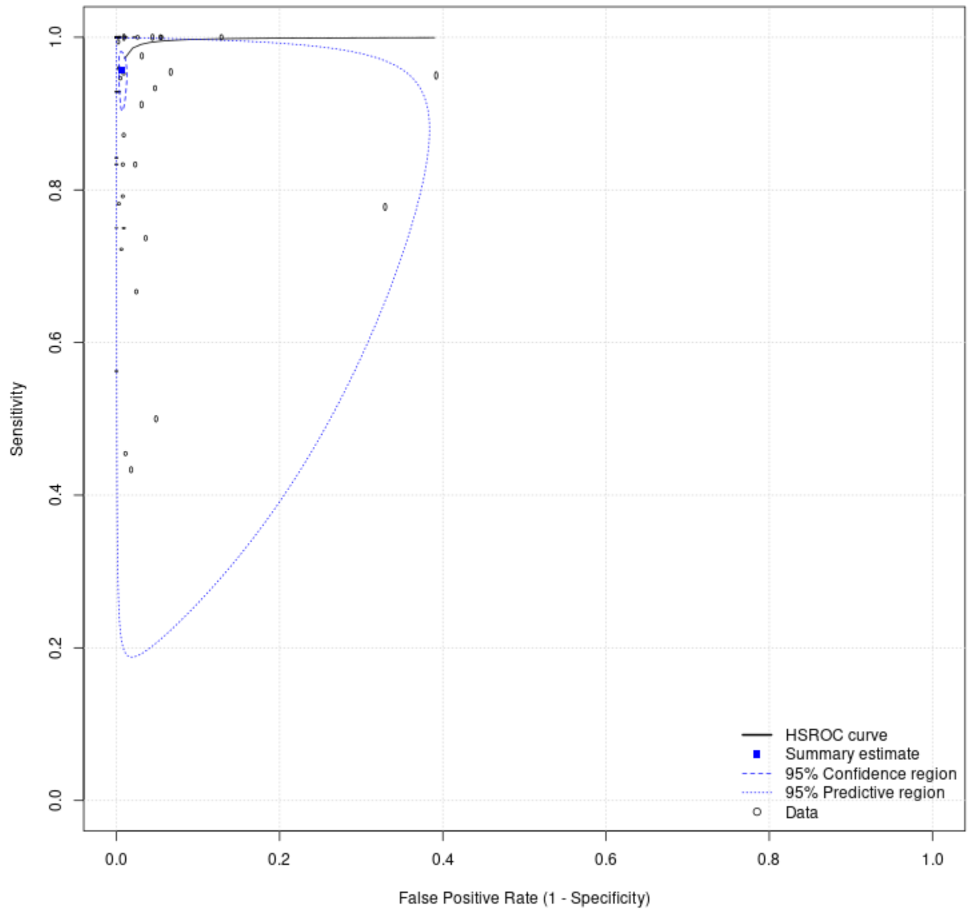

Figure S3. Hierarchical Summary ROC curve

3 **Model code for rjags-** Bayesian latent hierarchical (BHSROC) model

```
model_jags <- "model
{ resdev <- sum(resdevi[])          # Residual deviance
  for(i in 1:l) {
   cell[i,1:4] ~ dmulti(cell_prob[i,1:4],n[i])
   # Positive_Index - Positive_reference
  cell_prob[i,1] <- pi[i]*(  pp[i]    *  s2  ) + (1-pi[i])*(  pn[i]    * (1-c2))
   # Positive_Index - Negative_reference
 cell_prob[i,2] <- pi[i]*(  pp[i]    * (1-s2) ) + (1-pi[i])*(  pn[i]    *  c2)
   # Negative - Positive_reference
 cell_prob[i,3] <- pi[i]*( (1-pp[i]) *  s2  ) + (1-pi[i])*( (1-pn[i]) * (1-c2))
   # Negative_Index - Negative_reference
 cell_prob[i,4] <- pi[i]*( (1-pp[i]) * (1-s2) ) + (1-pi[i])*( (1-pn[i]) *  c2)
   ###  Conditional dependence:
 #cell_prob[i,1] <- pi[i]*(  pp[i]    *  s2    +covp[i]  ) + (1-pi[i])*(  pn[i]    * (1-c2) +covn[i] )
#cell_prob[i,2] <- pi[i]*(  pp[i]    * (1-s2) -covp[i] ) + (1-pi[i])*(  pn[i]    *  c2     -covn[i] )
 #cell_prob[i,3] <- pi[i]*( (1-pp[i]) *  s2    -covp[i] ) + (1-pi[i])*( (1-pn[i]) * (1-c2)  -covn[i] )
 # cell_prob[i,4] <- pi[i]*( (1-pp[i]) * (1-s2) +covp[i]  ) + (1-pi[i])*( (1-pn[i]) *  c2    +covn[i]
#####for reference standard's agar types
#cell[i,1:4] ~ dmulti(cell_prob[i,1:4],n[i])
 # Positive_Index - Positive_reference
#cell_prob[i,1] <- pi[i]*(  pp[i]    *  s2[ref[i]] ) + (1-pi[i])*(  pn[i]    * (1-c2[ref[i]]))
 # Positive_Index - Negative_reference
#cell_prob[i,2] <- pi[i]*(  pp[i]    * (1-s2[ref[i]]) ) + (1-pi[i])*(  pn[i]    *  c2[ref[i]])
# Negative - Positive_reference
# cell_prob[i,3] <- pi[i]*( (1-pp[i]) *  s2[ref[i]]  ) + (1-pi[i])*( (1-pn[i]) * (1-c2[ref[i]]))
# Negative_Index - Negative_reference
 # cell_prob[i,4] <- pi[i]*( (1-pp[i]) * (1-s2[ref[i]]) ) + (1-pi[i])*( (1-pn[i]) *  c2[ref[i]])
)
  # pp stands for : test under evaluation positive and reference standard positive (Crudely
referred to true positive rate or sensitivity)
# pn   stands for : test under evaluation positive and reference standard negative (Crudely
referred to false positive rate or 1-specificity)
 # Test under EVALUATION
   logit(pp[i]) <- (theta[i] + 0.5*alpha[i])/exp(beta/2)
   logit(pn[i]) <- (theta[i] - 0.5*alpha[i])*exp(beta/2)
```

```
 #theta BETWEEN study variation of the cut-off values
 theta[i] ~ dnorm(THETA,tau[1])
 #alpha --> variation in the distribution of tp in each study
 alpha[i] ~ dnorm(LAMBDA,tau[2])
# STUDY PREVALENCE PRIOR
 pi[i] ~ dbeta(1,1)
# Fitted values
rhat[i,1] <- cell_prob[i,1]*n[i]
rhat[i,2] <- cell_prob[i,2]*n[i]
rhat[i,3] <- cell_prob[i,3]*n[i]
rhat[i,4] <- cell_prob[i,4]*n[i]
resdevi[i] <- 2*(cell[i,1]*log(cell[i,1]/rhat[i,1])+cell[i,2]*log(cell[i,2]/rhat[i,2])
                  +cell[i,3]*log(cell[i,3]/rhat[i,3])+cell[i,4]*log(cell[i,4]/rhat[i,4]))
# STUDY-LEVEL SENSITIVITY AND SPECIFICITY OF TEST UNDER EVALUATION
 se[i] <- pp[i]
 sp[i] <- 1-pn[i]
 #  limits of covariance parameters
 #us[i]<-min(s2*(1-se[i]), (1-s2)*se[i])
 #uc[i]<-min(c2*(1-sp[i]), (1-c2)*sp[i])
 #ls[i]<- max(-(1-se[i])*(1-s2), -se[i]*s2)
 #lc[i]<- max(-(1-sp[i])*(1-c2), -sp[i]*c2)
  # prior distribution of transformed covariances on (0,1) range
 #covp[i]~dunif(ls[i],us[i])
 #covp[i]~dunif(0,us[i])
 #covn[i]~dunif(lc[i],uc[i])
 # covariance parameters as a fraction of their upper bound
#covp[i]<-0*us[i]
 #covn[i]<-0*uc[i]
# replace 0 above by other percentages, e.g. 0.05, 0.1, 0.2, ..for conditional dependence.
## Hierarchical structure over reference test(s)
        # hierarchical prior distribution over ref std properties
          # for (i in 1:l) {
          #logit(s2[i]) <- ls2[i]
         # ls2[i] ~ dnorm(mu.ls2, prec.ls2)
         # logit(c2[i]) <- lc2[i]
         # lc2[i] ~ dnorm(mu.lc2, prec.lc2)
        ##}#
```

```
            logit(s2) <- ls2
             ls2 ~ dnorm(mu.ls2,prec.ls2)
             logit(c2) <- lc2
            lc2 ~ dnorm(mu.lc2,prec.lc2)
##########random effects between reference types, fixed within study effects
#for (k in 1:3) {
#logit(s2[k]) <- ls2[k]
# ls2[k] ~ dnorm(mu.ls2[k], prec.ls2[k])
#logit(c2[k]) <- lc2[k]
# lc2[k] ~ dnorm(mu.lc2[k], prec.lc2[k])
}
 # HYPER PRIOR DISTRIBUTIONS FOR PARAMETERS DEFINING INDEX TEST
  THETA ~ dunif(-10,10)
  LAMBDA ~ dunif(-6,6)
  beta ~ dunif(-2,2)
  for(j in 1:2) {
    tau[j] <- pow(sigma[j],-2)
   sigma[j] ~ dgamma(4,2)
      }
####HIERARCHICAL PRIOR DISTRIBUTION OVER INDEX TEST SUBGROUPS
for(j in 1:ngroups){
              THETA[j] ~ dunif(-10,10)
              LAMBDA[j] ~ dunif(-6,6)
              beta[j] ~ dunif(-2,2)
              sigma1[j] ~ dgamma(4,2)
              tau1[j] <- pow(sigma1[j], -2)
              sigma2[j] ~ dgamma(4,2)
              tau2[j] <- pow(sigma2[j], -2)
              # Calculate Se and Sp for PCR j INSIDE the loop
              Se_PCR[j] <- 1/(1+exp((-THETA[j]-0.5*LAMBDA[j])/exp(beta[j]/2)))
              Sp_PCR[j] <- 1/(1+exp((THETA[j]-0.5*LAMBDA[j])*exp(beta[j]/2)))
  }
# HIERARCHICAL PRIOR DISTRIBUTION OVER REF STD PROPERTIES
mu.ls2 ~ dnorm(0,0.25)
mu.lc2 ~ dnorm(0,0.25)
#mu.ls2 ~ dnorm(0,0.25)T(-0.4,) # restricted prior
#mu.lc2 ~ dnorm(0,0.25)T(0.4,) # restricted prior
```

```
# informative prior ->Normal(lower and upper quantiles 84% and 98%)
 #mu.ls2 ~ dnorm(2.78, pow(0.57, -2))
#  informative prior->Normal(lower and upper quantiles 84% and 98%)
#mu.lc2 ~ dnorm(3.28, pow(1.03, -2))
prec.ls2 ~ dgamma(2,2)
prec.lc2 ~ dgamma(2,2)
S2<-1/(1+exp(-mu.ls2))
C2<-1/(1+exp(-mu.lc2))
#####REFERENCE AGAR TYPES
##for (k in 1:3) {
#mu.ls2[k] ~ dnorm(0,0.25)T(-0.4,)
#mu.lc2[k] ~ dnorm(0,0.25)T(0.4,)
#prec.ls2[k] ~ dgamma(2,2)
#prec.lc2[k] ~ dgamma(2,2)
#S2[k]<-1/(1+exp(-mu.ls2[k]))
#C2[k]<-1/(1+exp(-mu.lc2[k]))
#}
  # Pooled sensitivity and specificity
  Pooled_S<-1/(1+exp((-THETA-0.5*LAMBDA)/exp(beta/2)))
  Pooled_C<-1/(1+exp((THETA-0.5*LAMBDA)*exp(beta/2)))
   # Predicted sensitivity and specificity in a new study
  theta_new ~ dnorm(THETA,tau[1])
  alpha_new ~ dnorm(LAMBDA,tau[2])
  S.new <- 1/(1+exp(-(theta_new+0.5*alpha_new)/exp(beta/2)))
  C.new <- 1/(1+exp((theta_new-0.5*alpha_new)*exp(beta/2)))

}"
```

***4.* Model code for rjags-**Bayesian latent bivariate

```
model_jags <- "model {
  for(i in 1:l) {
     cell[i,1:4] ~ dmulti(cell_prob[i,1:4],n[i])
     # Positive_Index - Positive_reference
    cell_prob[i,1] <- pi[i]*( se[i]     * s2) + (1-pi[i])*( (1-sp[i])*(1-c2))
    # Positive_Index - Negative_reference
    cell_prob[i,2] <- pi[i]*( se[i]     * (1-s2)) + (1-pi[i])*( (1-sp[i]) *c2)
```

```
    # Negative - Positive_reference
    cell_prob[i,3] <- pi[i]*( (1-se[i]) * s2 ) + (1-pi[i])*( sp[i]*(1-c2))
    # Negative_Index - Negative_reference
    cell_prob[i,4] <- pi[i]*( (1-se[i]) * (1-s2)) + (1-pi[i])*( sp[i]*c2)
    # Hierarchical prior for multiplex PCR
    logit(se[i]) <- l1[i,1]
    logit(sp[i]) <- l1[i,2]
    l1[i,1:2] ~ dmnorm(mu[], T[,])
# Prior distribution on prevalence
    pi[i] ~ dbeta(1,1)
            }
# hierarchical prior distribution over ref std properties
logit(s2) <- ls2
 ls2 ~ dnorm(mu.ls2,prec.ls2)
 logit(c2) <- lc2
 lc2 ~ dnorm(mu.lc2,prec.lc2)
  # Hyper priors for multiplex
  mu[1] ~ dnorm(0,0.25)
  mu[2] ~ dnorm(0,0.25)
    T[1:2,1:2]<-inverse(TAU[1:2,1:2])
#### BETWEEN-STUDY VARIANCE-COVARIANCE MATRIX
            TAU[1,1] <- tau[1]*tau[1]
            TAU[2,2] <- tau[2]*tau[2]
            TAU[1,2] <- rho*tau[1]*tau[2]
            TAU[2,1] <- rho*tau[1]*tau[2]
#### prec = between-study precision in the logit(sensitivity) and logit(specificity)
            prec[1] ~ dgamma (2,0.5)
            prec[2] ~ dgamma(2,0.5)
            rho ~ dunif(-1,1)
#### BETWEEN_STUDY STANDARD DEVIATION
           tau[1]<-pow(prec[1],-0.5)
           tau[2]<-pow(prec[2],-0.5)
#### BETWEEN_STUDY VARIANCE
tau.sq[1] <- pow(tau[1], 2)
tau.sq[2] <- pow(tau[2], 2)
###PRIORS REFERENCE TEST:
#non informative priors
```

```
mu.ls2 ~ dnorm(0,0.25)I(-0.4,)
 mu.lc2 ~ dnorm(0,0.25)I(0.4,)
#restricted priors
#mu.ls2 ~ dnorm(0,0.25)I(-0.4,)
# mu.lc2 ~ dnorm(0,0.25)I(0.4,)
# informative priors
# mu.ls2 ~ dnorm(1.265, 1.56)   # center ~0.78, wide
# mu.lc2 ~ dnorm(1.265, 1.56)  # center ~0.985, moderate
prec.ls2 ~ dgamma(2,2)
 prec.lc2 ~ dgamma(2,2)
###SUMMARY SENSITIVITY AND SPECIFICITY OF REF.TEST
S2<-1/(1+exp(-mu.ls2))
 C2<-1/(1+exp(-mu.lc2))
#### SUMMARY SENSITIVITY AND SPECIFICITY OF INDEX TEST
Pooled_S<-1/(1+exp(-mu[1]))
Pooled_C<-1/(1+exp(-mu[2]))
#### PREDICTED SENSITIVITY AND SPECIFICITY OF INDEX TEST IN A FUTURE
STUDY
  l.new[1:2] ~ dmnorm(mu[],T[,])
  S_new <- 1/(1+exp(-l.new[1]))
  C_new <- 1/(1+exp(-l.new[2]))
 #inits#
  #monitor deviance pD
}"
```

References

1. Haddaway NR, Page MJ, Pritchard CC, McGuinness LA. PRISMA2020: An R package and Shiny app for producing PRISMA 2020-compliant flow diagrams, with interactivity for optimised digital transparency and Open Synthesis. Campbell Syst Rev [Internet]. 2022 Jun 1;18(2):e1230. Available from: https://doi.org/10.1002/cl2.1230
2. Ahmed AO, Abdelaziz AM, Rashed H-AG, Tolba M, Mahmoud AA. Evaluation of a multiplex polymerase chain reaction for the diagnosis of infectious diarrhea in intensive care unit patients in Upper Egypt. Egypt J Immunol [Internet]. 2024;31(1):1 – 9. Available from: https://www.scopus.com/inward/record.uri?eid=2-s2.0-85182546304&partnerID=40&md5=5bc6f80503b512799b56ab3080db0a3c
3. Bessède E, Delcamp A, Sifré E, Buissonnière A, Mégraud F. New Methods for Detection of Campylobacters in Stool Samples in Comparison to Culture. J Clin Microbiol [Internet]. 2011 Mar [cited 2021 Feb 3];49(3):941–4. Available from: http://www.ncbi.nlm.nih.gov/pubmed/21209172
4. Buchan BW, Olson WJ, Pezewski M, Marcon MJ, Novicki T, Uphoff TS, et al. Clinical evaluation of a real-time PCR assay for identification of salmonella, shigella, campylobacter (campylobacter jejuni and C. coli), and shiga toxin-producing escherichia coli isolates in stool specimens. J Clin Microbiol [Internet]. 2013 Dec 1 [cited 2021 Feb 27];51(12):4001–7. Available from: https://jcm.asm.org/content/51/12/4001
5. Buss SN, Leber A, Chapin K, Fey PD, Bankowski MJ, Jones MK, et al. Multicenter Evaluation of the BioFire FilmArray Gastrointestinal Panel for Etiologic Diagnosis of Infectious Gastroenteritis. Burnham C-AD, editor. J Clin Microbiol [Internet]. 2015 Mar;53(3):915–25. Available from: https://jcm.asm.org/content/53/3/915
6. Claas EC, Burnham C-AD, Mazzulli T, Templeton K, Topin F. Performance of the xTAG® gastrointestinal pathogen panel, a multiplex molecular assay for simultaneous detection of bacterial, viral, and parasitic causes of infectious gastroenteritis. J Microbiol Biotechnol [Internet]. 2013;23(7):1041 – 1045. Available from: https://www.scopus.com/inward/record.uri?eid=2-s2.0-84880654458&doi=10.4014%2Fjmb.1212.12042&partnerID=40&md5=43398228e268bd95ce7f2400d5dbdef4
7. Coupland LJ, Mcelarney I, Meader E, Cowley K, Alcock L, Naunton J, et al. Simultaneous detection of viral and bacterial enteric pathogens using the Seeplex® Diarrhea ACE detection system. Epidemiol Infect [Internet]. 2013;141(10):2111 – 2121. Available from: https://www.scopus.com/inward/record.uri?eid=2-s2.0-84883422539&doi=10.1017%2FS0950268812002622&partnerID=40&md5=1292423

7b5e7ee7ab2fe029ee74d0e81

8. Cybulski RJ, Bateman AC, Bourassa L, Bryan A, Beail B, Matsumoto J, et al. Clinical Impact of a Multiplex Gastrointestinal Polymerase Chain Reaction Panel in Patients With Acute Gastroenteritis. Clin Infect Dis [Internet]. 2018 Apr 25;67(11):1697–704. Available from: https://academic.oup.com/cid/advance-article/doi/10.1093/cid/ciy357/4985150

9. Deng J, Luo X, Wang R, Jiang L, Ding X, Hao W, et al. A comparison of Luminex xTAG® Gastrointestinal Pathogen Panel (xTAG GPP) and routine tests for the detection of enteropathogens circulating in Southern China. Diagn Microbiol Infect Dis [Internet]. 2015 Nov 1 [cited 2021 Feb 10];83(3):325–30. Available from: https://linkinghub.elsevier.com/retrieve/pii/S0732889315002886

10. Dror Ken, Pavlotzky E, Barak M, Dror SK, Pavlotzky E, Barak M, et al. Evaluation of the NanoCHIP® Gastrointestinal Panel (GIP) Test for Simultaneous Detection of Parasitic and Bacterial Enteric Pathogens in Fecal Specimens. Chang Y-F, editor. PLoS One [Internet]. 2016 Jul 22;11(7):e0159440. Available from: https://dx.plos.org/10.1371/journal.pone.0159440

11. Duong VT, Phat VV, Tuyen HT, Dung TTN, Trung PD, Minh P Van, et al. Evaluation of Luminex xTAG Gastrointestinal Pathogen Panel Assay for Detection of Multiple Diarrheal Pathogens in Fecal Samples in Vietnam. Richter SS, editor. J Clin Microbiol [Internet]. 2016 Apr;54(4):1094–100. Available from: https://jcm.asm.org/content/54/4/1094

12. Halligan E, Edgeworth J, Bisnauthsing K, Bible J, Cliff P, Aarons E, et al. Multiplex molecular testing for management of infectious gastroenteritis in a hospital setting: A comparative diagnostic and clinical utility study. Clin Microbiol Infect [Internet]. 2014;20(8):O460–7. Available from: http://dx.doi.org/10.1111/1469-0691.12476

13. Harrington SM, Buchan BW, Doern C, Fader R, Ferraro MJ, Pillai DR, et al. Multicenter Evaluation of the BD Max Enteric Bacterial Panel PCR Assay for Rapid Detection of Salmonella spp., Shigella spp., Campylobacter spp. (C. jejuni and C. coli), and Shiga Toxin 1 and 2 Genes. Gilligan PH, editor. J Clin Microbiol [Internet]. 2015 May [cited 2021 Apr 3];53(5):1639–47. Available from: https://jcm.asm.org/content/53/5/1639

14. Hu Q, Lyu DY, Shi X, Jiang Y, Lin Y, Li Y, et al. A Modified Molecular Beacons–Based Multiplex Real-Time PCR Assay for Simultaneous Detection of Eight Foodborne Pathogens in a Single Reaction and Its Application. Foodborne Pathog Dis [Internet]. 2014 Mar 1;11(3):207–14. Available from: http://www.liebertpub.com/doi/10.1089/fpd.2013.1607

15. Huang RSP, Johnson LC, Pritchard L, Hepler R, Ton TT, Dunn JJ. Performance of the

Verigene® enteric pathogens test, Biofire FilmArray™ gastrointestinal panel and Luminex xTAG® gastrointestinal pathogen panel for detection of common enteric pathogens. Diagn Microbiol Infect Dis. 2016 Dec 1;86(4):336–9.

16. Huang S-H, Lin Y-F, Tsai M-H, Yang S, Liao M-L, Chao S-W, et al. Detection of common diarrhea-causing pathogens in Northern Taiwan by multiplex polymerase chain reaction. Med (United States) [Internet]. 2018;97(23). Available from: https://www.scopus.com/inward/record.uri?eid=2-s2.0-85049196323&doi=10.1097%2FMD.0000000000011006&partnerID=40&md5=eda6656ed76c772afd00dbc6a0ecd0ec

17. Jo SJ, Kang HM, Kim JO, Cho H, Heo W, Yoo IY, et al. Evaluation of the biofire gastrointestinal panel to detect diarrheal pathogens in pediatric patients. Diagnostics [Internet]. 2022 Dec 24;12(1):34. Available from: https://www.mdpi.com/2075-4418/12/1/34

18. Kellner T, Parsons B, Chui L, Berenger BMBM, Xie J, Burnham C-ADC-AD, et al. Comparative Evaluation of Enteric Bacterial Culture and a Molecular Multiplex Syndromic Panel in Children with Acute Gastroenteritis. Ledeboer NA, editor. J Clin Microbiol [Internet]. 2019 Apr 10;57(6). Available from: https://jcm.asm.org/content/57/6/e00205-19

19. Khare R, Espy MJ, Cebelinski E, Boxrud D, Sloan LM, Cunningham SA, et al. Comparative evaluation of two commercial multiplex panels for detection of gastrointestinal pathogens by use of clinical stool specimens. J Clin Microbiol. 2014 Oct 1;52(10):3667–73.

20. Knabl L, Grutsch I, Orth-Höller D. Comparison of the BD MAX® Enteric Bacterial Panel assay with conventional diagnostic procedures in diarrheal stool samples. Eur J Clin Microbiol Infect Dis [Internet]. 2016 Jan 13;35(1):131–6. Available from: http://link.springer.com/10.1007/s10096-015-2517-4

21. Knoth C, Humphries R, Johnson JK, Patel A, Lima A, Silbert S, et al. Multicenter evaluation of BioCode GPP for syndromic molecular detection of gastrointestinal pathogens from stool specimens. J Clin Microbiol [Internet]. 2024;62(3). Available from: https://www.scopus.com/inward/record.uri?eid=2-s2.0-85187789437&doi=10.1128%2Fjcm.01545-23&partnerID=40&md5=ace8de0b92188c938effaf492739edf9

22. Koeffer J, Kolb M, Sorel O, Ulekleiv C, Feenstra JDM, Eigner U. Clinical performance evaluation of TAQPATH Enteric Bacterial Select Panel for the detection of common enteric bacterial pathogens in comparison to routine stool culture and other qPCR-based diagnostic tests. Microbiol Spectr [Internet]. 2024;12(1). Available from: https://www.scopus.com/inward/record.uri?eid=2-s2.0-

85182396335&doi=10.1128%2Fspectrum.03172-23&partnerID=40&md5=976ccb4de1136b5f487ef5e6e5607313

23. Köffer J, Frontzek A, Eigner U. Development and validation of a bacterial gastrointestinal multiplex RT-PCR assay for use on a fully automated molecular system. J Microbiol Methods [Internet]. 2023;210. Available from: https://www.scopus.com/inward/record.uri?eid=2-s2.0-85161012394&doi=10.1016%2Fj.mimet.2023.106754&partnerID=40&md5=efbe753150eebef80b1fff3f451ea5cc

24. Koo SH, Heng YX, Jiang B, Ng LSY, Sim DMF, Tan TY. Evaluation of the clinical sensitivity and specificity of the BD MaxTM Enteric Bacterial Panel for molecular detection of pathogens for acute gastroenteritis in the Singaporean population. J Microbiol Methods [Internet]. 2022 Jun 1 [cited 2022 Sep 8];197:106478. Available from: https://linkinghub.elsevier.com/retrieve/pii/S0167701222000732

25. Kosai K, Suzuki H, Tamai K, Okada Y, Akamatsu N, Ueda A, et al. Multicenter evaluation of Verigene Enteric Pathogens Nucleic Acid Test for detection of gastrointestinal pathogens. Sci Rep [Internet]. 2021 Feb 4 [cited 2022 Sep 8];11(1):3033. Available from: http://www.nature.com/articles/s41598-021-82490-z

26. Liu J, Gratz J, Amour C, Kibiki G, Becker S, Janaki L, et al. A Laboratory-Developed TaqMan Array Card for Simultaneous Detection of 19 Enteropathogens. J Clin Microbiol. 2013 Feb;51(2):472–80.

27. Liu J, Gratz J, Maro A, Kumburu H, Kibiki G, Taniuchi M, et al. Simultaneous Detection of Six Diarrhea-Causing Bacterial Pathogens with an In-House PCR-Luminex Assay. J Clin Microbiol [Internet]. 2012 Jan;50(1):98–103. Available from: https://jcm.asm.org/content/50/1/98

28. Martín A, Pérez-Ayala A, Chaves F, Lora D, Orellana MÁÁ. Evaluation of the multiplex PCR Allplex-GI assay in the detection of bacterial pathogens in diarrheic stool samples. J Microbiol Methods [Internet]. 2018 Jan;144:33–6. Available from: https://linkinghub.elsevier.com/retrieve/pii/S0167701217302890

29. McAuliffe G, Bissessor L, Williamson D, Moore S, Wilson J, Dufour M, et al. Use of the EntericBio Gastro Panel II in a diagnostic microbiology laboratory: challenges and opportunities. Pathology [Internet]. 2017 Jun;49(4):419–22. Available from: https://linkinghub.elsevier.com/retrieve/pii/S0031302516405581

30. Navidad JF, Griswold DJ, Gradus MS, Bhattacharyya S. Evaluation of Luminex xTAG Gastrointestinal Pathogen Analyte-Specific Reagents for High-Throughput, Simultaneous Detection of Bacteria, Viruses, and Parasites of Clinical and Public Health Importance. J Clin Microbiol. 2013 Sep;51(9):3018–24.

31. O'Leary J, Corcoran D, Lucey B. Comparison of the EntericBio Multiplex PCR

System with Routine Culture for Detection of Bacterial Enteric Pathogens. J Clin Microbiol [Internet]. 2009 Nov [cited 2021 Apr 25];47(11):3449–53. Available from: http://jcm.asm.org/

32. Onori M, Coltella L, Mancinelli L, Argentieri M, Menichella D, Villani A, et al. Evaluation of a multiplex PCR assay for simultaneous detection of bacterial and viral enteropathogens in stool samples of paediatric patients. Diagn Microbiol Infect Dis. 2014;79(2):149–54.

33. Pankhurst L, Macfarlane-Smith L, Buchanan J, Anson L, Davies K, O'Connor L, et al. Can rapid integrated polymerase chain reaction-based diagnostics for gastrointestinal pathogens improve routine hospital infection control practice? A diagnostic study. Health Technol Assess (Rockv) [Internet]. 2014 Aug;18(53):1–167. Available from: https://www.journalslibrary.nihr.ac.uk/hta/hta18530/

34. Park K, Shin B-M. Comparative evaluation of two molecular multiplex syndromic panels with acute gastroenteritis: Multiplex Syndromic Tests for Gastroenteritis. Diagn Microbiol Infect Dis [Internet]. 2024;109(1):116211. Available from: https://www.scopus.com/inward/record.uri?eid=2-s2.0-85186918813&doi=10.1016%2Fj.diagmicrobio.2024.116211&partnerID=40&md5=cd51596edfcc961e18011e5c903a84cc

35. Patel A, Navidad J, Bhattacharyya S. Site-specific clinical evaluation of the Luminex xTAG gastrointestinal pathogen panel for detection of infectious gastroenteritis in fecal specimens. J Clin Microbiol. 2014;52(8):3068–71.

36. Perry MD, Corden SA, Howe RA. Evaluation of the luminex xTAG Gastrointestinal Pathogen Panel and the Savyon Diagnostics Gastrointestinal Infection Panel for the detection of enteric pathogens in clinical samples. J Med Microbiol. 2014 Nov 1;63(11):1419–26.

37. Rintala A, Munukka E, Weintraub A, Ullberg M, Eerola E. Evaluation of a multiplex real-time PCR kit Amplidiag® Bacterial GE in the detection of bacterial pathogens from stool samples. J Microbiol Methods [Internet]. 2016 Sep;128:61–5. Available from: https://linkinghub.elsevier.com/retrieve/pii/S0167701216301816

38. Roy C, Robert D, Bénéjat L, Buissonnière A, Ducournau A, Mégraud F, et al. Performance Evaluation of the Novodiag Bacterial GE+ Multiplex PCR Assay. Onderdonk AB, editor. J Clin Microbiol [Internet]. 2020 Sep 22;58(10). Available from: https://journals.asm.org/doi/10.1128/JCM.01033-20

39. Tilmanne A, Martiny D, Quach C, Wautier M, Vandenberg O, Lepage P, et al. Enteropathogens in paediatric gastroenteritis: comparison of routine diagnostic and molecular methods. Clin Microbiol Infect [Internet]. 2019;25(12):1519 – 1524. Available from: https://www.scopus.com/inward/record.uri?eid=2-s2.0-

85071869863&doi=10.1016%2Fj.cmi.2019.07.021&partnerID=40&md5=efd983623e1cfaee2e7640876fcc1cdb

40. Van Lint P, De Witte E, De Henau H, De Muynck A, Verstraeten L, Van Herendael B, et al. Evaluation of a real-time multiplex PCR for the simultaneous detection of Campylobacter jejuni, Salmonella spp., Shigella spp./EIEC, and Yersinia enterocolitica in fecal samples. Eur J Clin Microbiol Infect Dis [Internet]. 2015 Mar 19;34(3):535–42. Available from: http://link.springer.com/10.1007/s10096-014-2257-x

41. Wiemer D, Loderstaedt U, von Wulffen H, Priesnitz S, Fischer M, Tannich E, et al. Real-time multiplex PCR for simultaneous detection of Campylobacter jejuni, Salmonella, Shigella and Yersinia species in fecal samples. Int J Med Microbiol [Internet]. 2011 Nov;301(7):577–84. Available from: https://linkinghub.elsevier.com/retrieve/pii/S1438422111000658

42. Wohlwend N, Tiermann S, Risch L, Risch M, Bodmer T, Bodmer labormedizinisches zentrum Risch T, et al. Evaluation of a Multiplex Real-Time PCR Assay for Detecting Major Bacterial Enteric Pathogens in Fecal Specimens: Intestinal Inflammation and Bacterial Load Are Correlated in Campylobacter Infections. J Clin Microbiol. 2016 Sep;54(9):2262–6.

43. Yoo J, Park J, Lee HK, Yu JK, Lee GD, Park KG, et al. Comparative evaluation of Seegene allplex gastrointestinal, luminex XTAG gastrointestinal pathogen panel, and BD Max enteric assays for detection of gastrointestinal pathogens in clinical stool specimens. Arch Pathol Lab Med [Internet]. 2019;143(8):999 – 1005. Available from: https://www.scopus.com/inward/record.uri?eid=2-s2.0-85069806900&doi=10.5858%2Farpa.2018-0002-OA&partnerID=40&md5=f6fc826e336ddb8425ba6559d6b79a60

44. Zhang C, Niu P, Hong Y, Wang J, Zhang J, Ma X. A probe-free four-tube real-time PCR assay for simultaneous detection of twelve enteric viruses and bacteria. J Microbiol Methods [Internet]. 2015;118:93 – 98. Available from: https://www.scopus.com/inward/record.uri?eid=2-s2.0-84941194340&doi=10.1016%2Fj.mimet.2015.08.023&partnerID=40&md5=6e7f59cb05fe3d39c81996b86b9f5c9e

45. Zhang J, Guan H, Zhao W, Zhang H, Wang W, Ling X, et al. Evaluation of the BioFire FilmArray Gastrointestinal Panel and Real-Time Polymerase Chain Reaction Assays for the Detection of Major Diarrheagenic Pathogens by a Multicenter Diarrheal Disease Surveillance Program in China. Foodborne Pathog Dis [Internet]. 2019 Nov 1 [cited 2021 Mar 28];16(11):788–98. Available from: https://www.liebertpub.com/doi/10.1089/fpd.2019.2642
